# Ultrasound Guided Continuous Sciatic Nerve Block for Acute Herpetic Neuralgia

**DOI:** 10.1155/2019/7948282

**Published:** 2019-07-02

**Authors:** Thierry C. Bagaphou, Domenico Santonastaso, Eleonora Gargaglia, Lucia Norgiolini, Cinzia Tiburzi, Stefano Cristallini, Vittorio Cerotto, Fabio Gori

**Affiliations:** ^1^Section of Anesthesia, Intensive Care and Pain Medicine, Department of Emergency and Urgency, Città di Castello Hospital, Città di Castello, Via L. Angelini 10, 06012 Perugia, Italy; ^2^Section of Anesthesia and Intensive Care, AUSL Romagna, Local Health Authority, M. Bufalini Hospital, Viale Ghirotti 286, Cesena, Italy; ^3^Section of Anesthesia, Analgesia and Intensive Care, Department of Surgical and Biomedical Sciences, Santa Maria della Misericordia Hospital, Perugia, Italy; ^4^Section of Anesthesia, Intensive Care and Pain Medicine 1, Santa Maria della Misericordia Hospital, Perugia, Italy

## Abstract

Herpes Zoster (HZ) is the reactivation of a well-known viral disease which manifests itself with painful skin lesions. An effective analgesic method during the acute phase of HZ can contribute to decrease the incidence of postherpetic neuralgia (PHN) by reducing neural sensitization. Sciatic nerve block (SNB) is useful in the management of distal lower extremity pain sustained by the sciatic nerve. We describe our experience with a continuous ultrasound guided subgluteus sciatic nerve block in a patient with herpetic neuralgia- (HN-) related refractory acute left leg pain.

## 1. Introduction

Herpes zoster (HZ) is the reactivation of the Varicella Zoster virus (VZV) which accesses the sensory ganglia during primary infection and typically causes painful skin lesions. Although the vesicular rash disappears after a few weeks the pain may persist, resulting in postherpetic neuralgia (PHN) [1]. PHN is a condition of persistent neuropathic pain affecting about 50% of the over sixty population [2]. In most cases PHN follows the dermatomal distribution of the previous HZ skin rash, along the innervation of one or more spinal nerves, resulting in a radiculopathy [3]. Intercostal radiculopathy is the most frequent form of PHN, along with trigeminal nerve involvement, whereas the lumbosacral form is less frequent [4]. Typical symptoms of PHN include burning, stabbing pain associated with dysesthesia, and allodynia in the interested area. There is strong evidence that the aggressive treatment of herpetic neuralgia during the acute phase with antiviral drugs and multimodal analgesia can decrease the incidence of PHN by reducing neural sensitization [5]. Neuraxial treatment with epidural blocks, associated with antiviral therapy, may be used to reduce the acute pain caused by HZ to prevent the PHN [6]. Recent studies have also shown promising results with peripheral nerve blocks [7–10].

This case report describes the use of a continuous ultrasound-guided sciatic nerve block (US-SNB), with programmed intermittent boluses (PIB) technique, in a patient with Herpetic neuralgia- (HN-) related refractory acute left leg pain.

## 2. Case Report

In September 2018, a 62-year-old man was admitted to our Internal Medicine department with severe pain and a varicelliform skin eruption in the lower left limb which, appeared at least 20 days earlier.

He was affected by hypothyroidism and chronic lymphatic leukemia (stage II according to RAI and Binet) and was undergoing in outpatient chemotherapy treatment.

He complained of intense acute pain level 10 on the Numeric Rating Scale (NRS: ranging from 0: no pain to 10: the most intense pain ever experienced) along the entire length of the left lumbar root ganglia L5-S1. He presented vesicular lesions alternated with itchy scabs across the left gluteus muscle, with more intense lesions in the lower third and the rear of thigh, the popliteal fossa, and the back of leg up to the ankle. The patient also reported poor quality sleep for at least 15 days due to the continuous pain. Upon clinical assessment HZ with acute HN was diagnosed, Oral Antiviral, Pregabalin (75 mg x 2/day), Oxycodone/naloxone (10 mg/5 mg x2/day), and Paracetamol (1 g as needed) were prescribed.

After 5 days of hospitalisation and, almost, 2 weeks of therapy, the patient continued to reported NRS=10; therefore the attending physician requested an assessment from our department of Pain Medicine. After obtaining informed consent, the anaesthesiologist performed an epidural antalgic block at L3-L4 level with levobupivacaine 0.25% (4 ml), resulting in slight relief (NRS=8) for the next 6 hours only.

After 5 days of persistent level 8-9 pain on NRS with associated poor sleep quality, a new evaluation was requested. The patient also developed nausea, dizziness, and mental confusion as a result of drug therapy. After obtaining informed consent, we decided to perform a continuous US-SNB with subgluteus approach because the vesicular lesions had since dried up.

We decided to place the catheter on the sciatic nerve, at the subgluteal level, because the NRS 8-9 pain was more intense in the lower third of the thigh, on the popliteal region, and in the back of the leg.

With the patient in a semiprone position with the block limb uppermost, after skin disinfection with chlorhexidine 2%, we placed a low frequency convex probe, 2- 5 MHz over the subgluteal region in a transverse plan, between the greater trocantere, and the ischial tuberositis. We initially performed a systematic anatomical survey of structures from superficial to deep and medial to lateral; we identified the gluteus maximus muscle (GMm), the quadratus femoris muscles (QFm), the greater trochanter (GT) laterally, and the ischial tuberosity (IT) medially and we recognized the sciatic nerve as a hyperechoic and lip shaped oval structure inside a space lined by a hyperechoic margin formed by the surrounding muscles. Under aseptic precaution, we inserted a 18 G Tuohy needle (Kit Sonolong Curl Echo, Pajunk, ) using in the plane approach, from the lateral side to the medial part of the thigh, and we pushed the needle tip forward reaching the perineural space close to the sciatic nerve. Final needle position was confirmed by injecting 5 ml of saline solution and observing distention of the perineural space. A 20 G, multihole catheter with 6 lateral holes was then inserted under real time ultrasound guidance and advanced 3 cm over the needle tip into the perineural space. The needle was then withdrawn and a real time assessment of saline solution spread confirmed optimal catheter positioning.

After securing the catheter with Tegaderm transparent dressing, we administered a bolus of 10 ml lidocaine 2%, resulting in rapid pain relief at levels 0-1 on NRS associated with a motor block of the left leg and a foot extension deficit. Consequently, we started a PIB infusion of levobupivacaine 0.125%, 6 ml every 2 hours with possible additional boluses controlled by the patient (additional bolus 6 ml, one hour after PIB) using a CADD-Solis pump through the catheter.

After six hours, pain intensity was 0-2 on the NRS, nine hours after the procedure, andNRS was evaluated as 0 and the patient was able to sleep through the night. PIB were administered for 36 hours and then suspended because the pain was totally controlled, but the catheter was left in place. Drug therapy with pregabalin 75 mg x 2/day only was resumed.

Nine days after the procedure the patient was discharged with NRS=0, no motor block, a good sleep quality, and a significant reduction in pharmacological side effects.

At the one week, 1 month, 3 months, and 6 months of follow-up the patient was satisfied, pain-free, and enjoying good quality sleep. At the last check it was decided to lower and suspend pregabalin.

## 3. Discussion

Most cases of HZ affect elderly or immunocompromised patients. HZ strikes peripheral nerves and/or central roots causing pain, dysesthesia, and dysautonomia. Neuralgia is one of the most feared complications as it causes marked disability, compromising patients' quality of life [1]. Independent predictive factors for the development of PHN include advanced age, severe pain, and the simultaneous involvement of more than 2 dermatomes [5]. Early treatment with antivirals may reduce pain severity and the onset of PHN [2].

Although effectives, neuroaxial blocks (lumbar, thoracic or cervical) are may be associated with potentially complications, and cannot indicate in all patients especially those disorders coagulation, anticoagulants or serious comorbidities [4–6].

Ultrasound guided peripheral nerve blocks may become a valid alternative to neuraxial blocks in case of contraindications or ineffectiveness of the latter, as was the case of our patient [7–9].

The US-SNB in the subgluteal region carried out in our patient enabled the rapid disappearance of pain and dysesthesia and a significant improvement of sleep quality, proving to be a valid and safe alternative to neuraxial block. A long-acting local anaesthetic infusion, using the P.I.B technique has been applied for our patient, also because literature contains several studies demonstrating that local anaesthetic administrated using P.I.B and continuous infusion technique and provides similar pain relief [11]. A P.I.B infusion technique with possible additional bolus (6 ml after 1 hour, with 1 hour lookout), the patient has never delivered an additional bolus. We used a multihole catheter to optimize anaesthetic local spread.

Patients with PHN may present complex situations and, in addition to therapeutic guidelines, they need to be individually managed, according to clinical situations and comorbidities. We should also emphasize the importance of quick and effective intervention in preventing pain centralization. If pharmacological therapy proves to be ineffective, or if pain actually worsens as in our report, locoregional analgesic technique may be decisive. Although the epidural block remains the most widespread approach in such cases [12], and peripheral nerve blocks are becoming increasingly important as alternative or synergistic techniques, especially when a PIB technique is feasible.

There are few reports of peripheral nerve blocks used to treat PHN. The use of a PECS II block and of an erector spinae (ESP) block has been reported in cases of HZ-related acute pain or PHN, where neuraxial block was ineffective or impossible to perform [7–10]. A randomized controlled trial demonstrated that repetitive paravertebral anaesthetic block in combination with oral standard treatment significantly reduced the incidence of PHN compared to standard treatment alone [13]; however, only one case report described the successful application of a continuous paravertebral block using a paravertebral catheter to treat a case of PHN [14].

Our experience confirms that peripheral nerve blocks can be an excellent option in cases of Acute HN nonresponsive to standard treatments, even when the sciatic nerve is involved, validating the use of a catheter to perform a continuous peripheral nerve block to manage difficult cases with intractable pain. The recommended duration for infusion of anaesthetic local in continuous peripheral nerve blokade are not weel defined in litterature, must not exceed 48h–72h, depending on the patient's comorbidities, extreme age, renal faillure, hepatic faillure, low cardiac agitation, increased risk of local anesthetic toxicity due to alteration of absorption and elimination and therefore the concentration of A.L can remain high in the circolation for longer periods [11–16]. Our experience also confirms that this procedure has the merit of having good results not only in the short term, but also in the long term [15], while also helping to prevent post herpetic neuralgia ( PHN).

Future studies are needed to determine the real efficacy of peripheral nerve blocks compared to other treatment methods.
